# First Report of *Coffea arabica* Fruit Rot Disease Caused by *Fusarium coffeibaccae* in China

**DOI:** 10.3390/jof12030191

**Published:** 2026-03-06

**Authors:** Rui Wang, Yunjin Shi, Jin Xu, Wen Fu, Xiahong He, Xin Hao, Jie Chen

**Affiliations:** 1 Yunnan Provincial Key Laboratory for Conservation and Utilization of In-forest Resource, Southwest Forestry University, Kunming 650224, China; 2 Plant Conservation and Quarantine Station of Yunnan Province, Kunming 650034, China

**Keywords:** multilocus phylogeny, Koch’s postulates, pathogen

## Abstract

*Coffea arabica*, a popular beverage ingredient, is prized for its rich chemical composition, which has demonstrated significant positive effects in terms of antioxidant, anti-inflammatory, neuroprotective, and metabolic health. In November 2024, fruit rot with a 15% incidence was observed on *C. arabica* in Menglian city, Yunnan province, China. Symptoms began as irregular black spots that turned necrotic, wrinkled, and cracked. Fungal isolates from lesions showed morphological characteristics consistent with *Fusarium coffeibaccae*. Morphological data were supplemented with phylogenetic analyses based on three loci (*ITS*, *TEF1-α*, *RPB2*), and sequences were deposited in GenBank as for *ITS* (PV211189 and PV211190), *TEF1-α* (PQ867811 and PQ867812), and *RPB2* (PV261064 and PV261065). Koch’s postulates were fulfilled on attached fruits. After 17 days at 25 °C with 70% humidity, typical rot symptoms appeared on inoculated fruits, while controls remained symptom-free. This is the first report of *C. arabica* fruit rot caused by *F. coffeibaccae* in China. This study aims to identify the aetiological agent of recently observed coffee fruit rot in Yunnan and to characterize *F. coffeibaccae*. It provides the first baseline data for targeted monitoring and sustainable control of *F. coffeibaccae*-mediated fruit rot in China’s expanding coffee sector.

## 1. Introduction

Coffee is a significant cash crop in tropical regions, yielding substantial economic benefits for growers [1]. Also, research has demonstrated that moderate coffee consumption may slow the ageing process and contribute to improved cardiovascular and hepatic function [2,3]. Yunnan, one of China’s primary coffee-producing provinces, has a climate similar to that of coffee’s ancestral homeland and today’s leading growing regions. Its unique combination of high altitude and low latitude creates an exceptionally favorable environment for growing Arabica [4]. The coffee produced in Yunnan has a unique taste, mellow and slightly fruity [5]. A suspected fungal pathogen causing fruit rot in Yunnan’s coffee-growing regions has emerged as a potential threat to the sustainable development of this distinctive agricultural industry, though the taxonomic status of the causative fungus remains unconfirmed. Accurate identification of this pathogen not only helps prevent the misuse of pesticides but also supports the sustainable production of high-quality coffee berries, thereby providing a scientific foundation for effective disease management strategies.

Coffee fruit rot, a disease that severely affects both the yield and quality of coffee fruits, not only reduces the fruit’s commercial value but can also inhibit tree growth and potentially cause plant death. This results in substantial economic losses for the coffee industry. Coffee fruit rot was first reported in Kenya in 1922, caused by *Colletotrichum* sp., resulting in losses of up to 75% [6]. Currently, species associated with the occurrence of coffee fruit rot include *Hypothenemus hampei* (coffee berry borer, CBB), *Colletotrichum* spp., and *Fusarium* spp. In 2024, Puerto Rico reported for the first time that coffee berry disease was associated with *Fusarium* spp. [7]. The coffee berry borer acts as a vector for fungal pathogens such as *Fusarium* spp. and *Colletotrichum* spp., and its boring activity facilitates their entry into the berries, leading to internal decay and external canker formation [8]. Collectively, these biotic interactions depress farm-gate value, accelerate tree decline, and threaten the ecological and economic stability of coffee-growing landscapes.

*Fusarium* spp. is a globally distributed genus of fungi and ranks among the most challenging and significant diseases to control in agricultural production. Its detrimental effects are widespread; reports indicate that *Fusarium* spp. can cause various plant diseases, including root rot [9], stem rot [10], blossom end rot [11], and fruit rot [12]. These conditions adversely impact both yield and quality, inflicting substantial economic losses upon agriculture. For coffee, an essential tropical cash crop, the damage caused by *Fusarium* sp. is particularly significant. As one of the world’s leading coffee producers, Brazil has identified *Fusarium* species associated with coffee wilt disease, including *F. xylarioides*, *F. decemcellulare*, and *F. solani* [13,14]. These species can cause wilting and death in coffee, severely impacting yield and quality. In sub-Saharan African coffee-growing regions, recurrent outbreaks of coffee wilt disease caused by *F. xylarioides* have inflicted significant damage on local coffee economies, with Ethiopia, for example, experiencing severe yield reductions [15]. Research in Puerto Rico has also demonstrated that multiple *Fusarium* sp. are associated with coffee fruit disease in the region [7], further highlighting the threat posed by *Fusarium* sp. to coffee production. Therefore, we hypothesize that *Fusarium* species are likely causative agents of coffee berry disease in Yunnan’s coffee-growing regions in China. This study aims to isolate and purify potential *Fusarium* pathogens associated with coffee berry disease in China, confirm their pathogenicity through inoculation experiments, and determine the taxonomic identity of the pathogen by integrating morphological characteristics with molecular analyses.

## 2. Materials and Methods

### 2.1. Sample Collection, Fungus Isolation and Monoconidial Purification

In November 2024, diseased fruits were collected with 4-year-old *C. arabica* in Menglian, Yunnan, China (99°40′ E, 22°22′ N). A four-point survey was conducted in the orchard, covering a total of 50 trees. From each tree, 10 fruits were selected in four directions (east, west, south, and north). This method was used to investigate the incidence of fruit diseases. Incidence rate (%) (proportion of diseased fruits) = (Fruits with symptoms/Total number of surveyed fruits) × 100. A total of 40 samples from various randomly selected fruits were subjected to microscopic examination (Olympus CX33, ×100 magnification) (Olympus Corporation, Tokyo, Japan). The single-spore isolation method was used for pathogen purification and characterization [16]. Symptomatic fruits collected from the field were surface-sterilized in 75% ethanol for 30 s and rinsed 3 times with sterile distilled water. Under microscopic examination (Olympus CX33, Olympus Corporation, Tokyo, Japan), 10 conidia were lifted from diseased coffee fruits with a sterile needle and transferred to the potato dextrose agar plate (PDA; 200 g potato, 20 g glucose, 17 g agar L^−1^, natural pH; Beijing Aoboxing, Beijing, China) [17,18]. Once pure colonies appeared, they were successively sub-cultured three times to ensure purity, then maintained on PDA plate at 25 °C. Two negative controls were incubated under identical conditions to detect contamination. The growth rate was assessed on PDA at 25 °C in darkness. A 5-mm mycelial plug, taken from the colony margin, was centrally positioned on 90-mm plates, with three replicates for each isolate. Colony diameters were recorded in two perpendicular directions at 24, 48, 72, and 96 h after inoculation. The radial growth rate, expressed in mm/day, was determined through linear regression of the colony radius against time. The representative strains (SWFU10 and SWFU11) were deposited on slant medium at 4 °C in Southwest Forestry University.

### 2.2. Morphological Identification of the Fungus and Morphometric Analysis

Observations were made on pure cultures on PDA plates. Morphological identification was carried out based on colony texture and color as well as conidial morphology, conidial size and conidiophore status with reference to the literature [19]. Observations were conducted using a microscope (Olympus CX33, ×400 magnification, Olympus Corporation, Tokyo, Japan).

### 2.3. DNA Extraction, Polymerase Chain Reaction and Sequencing

To extract total DNA, mycelium was scraped from the pre-purified strains. The resulting mycelium was ground into powder in liquid nitrogen. Genomic DNA was extracted from the 1 g mycelium using a Plant Genomic DNA Kit (Tiangen, Beijing, China; catalog number DP305) according to the manufacturer’s instructions. DNA integrity was checked by 0.8% gel electrophoresis. Using the primers (Table 1), the translation elongation factor 1α (*TEF1-α*), RNA polymerase second largest subunit (*RPB2*), and internal transcribed spacer region of the nrDNA regions (*ITS*) were amplified. These three genes were cloned and sequenced. PCR amplification was performed in a 50 μL reaction mixture containing 1× PCR buffer (10 mM Tris-HCl, pH 8.9; 50 mM KCl; 1.5 mM MgCl_2_), 200 μM of each dNTP, 1.0 μM of each primer, 2.5 U TaKaRa Taq DNA polymerase (TaKaRa, R001A, TaKaRa Bio Inc., Shiga, Japan), and 50–100 ng genomic DNA template. PCR conditions were as follows: 95 °C for 5 min; 35 cycles at 95 °C for 30 s, 53 or 56 °C for 30 s, and 72 °C for 1 min; followed by 10 min at 72 °C. All primers were synthesized by Sangon Biotech Co., Ltd. (Shanghai, China). The amplification products were detected for their integrity through 0.8% gel electrophoresis and sequenced using the Sanger method by Sangon Biotech Co., Ltd. (Shanghai, China).

### 2.4. DNA Sequence Analysis

The obtained sequences were compared with those deposited in GenBank using BLASTN (https://blast.ncbi.nlm.nih.gov/Blast.cgi?PROGRAM=blastn&PAGE_TYPE=BlastSearch&LINK_LOC=blasthome, accessed on 13 February 2026)). For phylogenetic analyses, reference sequences (Table 2) were retrieved from GenBank. Maximum-likelihood (ML) analysis based on concatenated gene sequences was conducted in MEGA 12 with 1000 bootstrap replications to assess branch support.

Bayesian Phylogenetic: All sequence data were imported into the R statistical environment (R v4.5.1). Sequence alignments in FASTA format were read using the read.dna() function implemented in the ape package (v5.8). To facilitate downstream Bayesian phylogenetic inference, sequence files were converted to NEXUS format using the write.nexus.data() function within the same package. The resulting NEXUS files were used as input for phylogenetic analyses. Bayesian phylogenetic analyses were conducted using MrBayes (v3.2.7a), which was executed from within R via the system() function. Nucleotide substitution was modeled under the General Time Reversible model with gamma-distributed rate heterogeneity among sites (GTR + G). Model priors were specified as follows: base frequencies were assigned a Dirichlet prior (Dirichlet [1,1,1,1]), substitution rate parameters were assigned a Dirichlet prior (Dirichlet [1,1,1,1,1,1]), and the gamma shape parameter was assigned an exponential prior. Markov chain Monte Carlo (MCMC) simulations were run for 10,000,000 generations with four parallel chains, sampling every 1000 generations. Convergence was assessed using standard diagnostics implemented in MrBayes. The initial 25% of sampled trees were discarded as burn-in. A 50% majority-rule consensus tree was generated from the remaining posterior samples, and posterior probabilities (PP) were calculated to assess nodal support.

Phylogenetic trees were visualized and annotated in R using the ggtree (v3.12.0) and treeio (v1.28.0) packages. The consensus tree generated by MrBayes was imported using the read.mrbayes() function, which retained branch length and posterior probability information.

### 2.5. Pathogenicity Test

Fresh green plants with healthy coffee fruits (*C. arabica*) were collected in Menglian, Yunnan. To verify pathogenicity, healthy coffee fruits were inoculated with a conidial suspension (1 × 10^6^ conidia/mL) of SWFU10 with a hemocytometer, a representative isolate from the ten strains obtained in this study, while control plants were treated with sterile water attached fruits on the plant. The healthy fruits were surface-disinfested with 70% ethanol, rinsed twice with sterile distilled water, and blotted dry with sterile filter paper. Then a micro-syringe was used to inject 10 µL of spore suspension beneath the epidermis of each berry to simulate wound invasion; control berries received an equal volume of sterile water. Twenty fruits were inoculated per treatment. Each inoculated fruit-bearing branch was subjected to a two-stage incubation protocol. First, it was placed in a humidified polyethylene bag (≈75% RH) for 72 h. Subsequently, after bag removal, it was cultivated in an environmental chamber set at 25 °C with a 12 h photoperiod and a light intensity of 7000 lux [24]. The development of lesions on coffee fruits in both the treated and control groups was monitored and recorded after 17 days. To fulfill Koch’s postulates, the pathogen was re-isolated from the newly developed necrotic lesions using the single-spore isolation method to obtain a pure culture. The re-isolated strain (SWFU11) was then systematically compared with the original inoculum through microscopic examination of conidial morphology and colony characteristics, as well as sequence analysis of the *ITS*, *TEF-1α*, and *RPB2* gene regions.

## 3. Results

### 3.1. Fungus Isolation and Monoconidial Purification

**Figure 1:**
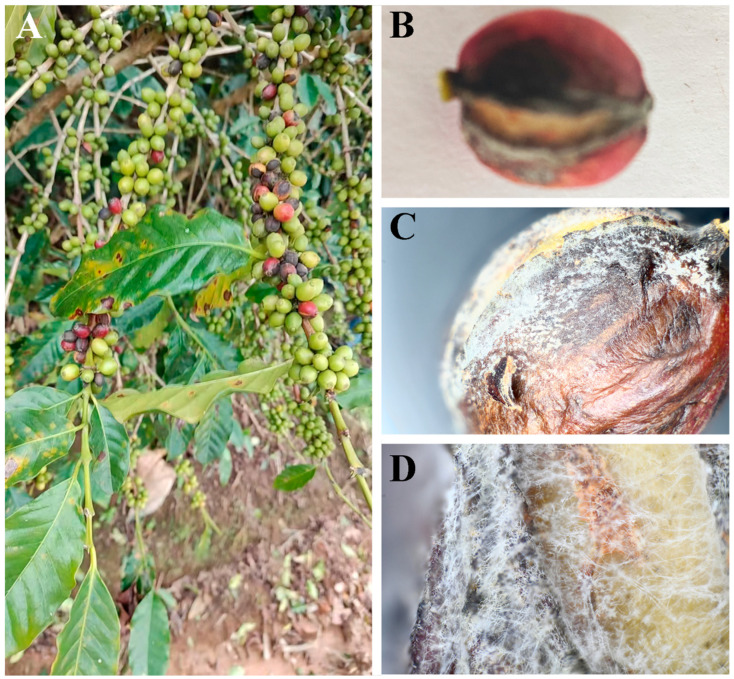
Symptoms of coffee fruit rot observed in Yunnan Province. (**A**) Diseased fruits in the field showing typical fruit rot symptoms; (**B**–**D**) Progressive symptom development on infected fruits: fruits exhibited premature ripening, followed by a color change from red to brown, and eventually became black, necrotic, wrinkled, and cracked while remaining attached to the branches.

Symptoms of fruit rot were detected on *C. arabica*, with a disease incidence of 15% (Figure 1A). Initially, the fruits ripened prematurely. Then, they turned red to brown (Figure 1B). Eventually, the fruits became black and necrotic, exhibiting wrinkling and cracking but remaining attached (Figure 1C,D). Ten strains were isolated by single-spore isolation and cultured on PDA, with one representative strain designated as SWFU10. Radial growth rate on PDA at 25 °C was determined for each isolate (*n* = 3) by measuring colony diameter at 24 h intervals for 4 d. Growth rates ranged from 12.5 to 15.8 mm/d (mean ± SD = 14.2 ± 1.1 mm/d). The representative strain SWFU10, used for phylogenetic and pathogenicity studies, grew at 14.5 ± 0.3 mm/d.

### 3.2. Cultural and Morphological Characteristics

**Figure 2:**
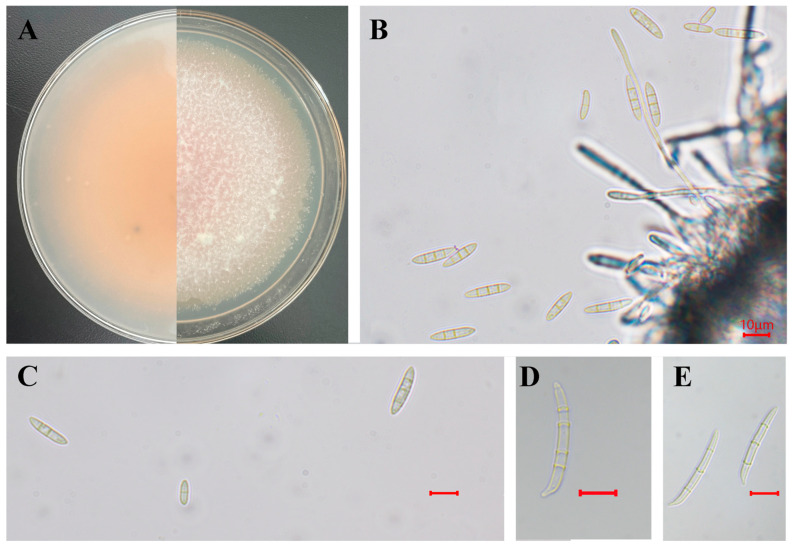
Morphology of *F. coffeibaccae.* SWFU10 and SWFU11. (**A**) Colonies on PDA; (**B**,**C**) Conidiophores and conidia; (**D**,**E**) Macroconidia. scale bar = 10 μm.

The SWFU10 and SWFU11 colonies were white in the beginning and completely covered 60 mm PDA plates; the reverse side was light orange. The mycelium was dense and cottony (Figure 2A). Conidiophores were simple or branched, apically producing conidia. Microconidia were fusiform to obovoid, straight to slightly curved, with a flattened base, 0 septate conidia, 9.3–14.2 × 3.1–4.2 µm (av. 13.91 × 3.65 µm); 1 septate conidia: 16.93–24.72 × 3.39–4.51 μm (av. 20.31 × 4.02 μm) (*n* = 50); 2-septate conidia: 21.25–24.25 × 3.8–4.8 μm (av. 22.4 × 4.55 μm) (*n* = 50) (Figure 2B,C). Macroconidia were straight to slightly curved, basal cell papillate to poorly developed, foot shaped, hyaline, smooth and thin walled, 3 septate conidia, 24.27–39.58 × 3.5–5.24 μm (av. 35.35 × 4.29 μm); 4 septate conidia, 33.69–48 × 3.56–4.9 µm (av. 44.23 × 4.15 μm) (*n* = 20) (Figure 2D,E). The morphological features were consistent with those of *F. coffeibacca*.

### 3.3. Phylogenetic Analyses

**Figure 3:**
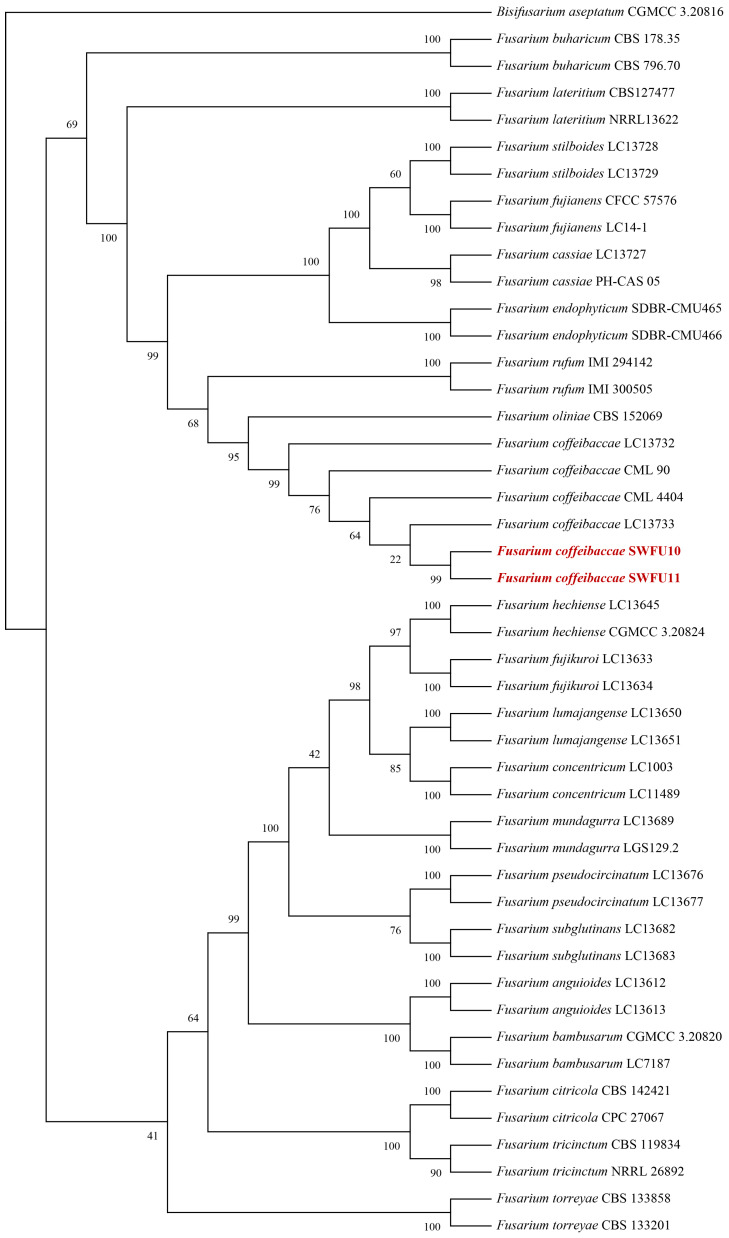
Phylogenetic tree from maximum likelihood analysis of combined *ITS*, *TEF1-α* and *RPB2* sequences. The values above the branches indicate bootstrap clustered test values (1000 replicates). Newly obtained sequences in this study are in red.

**Figure 4:**
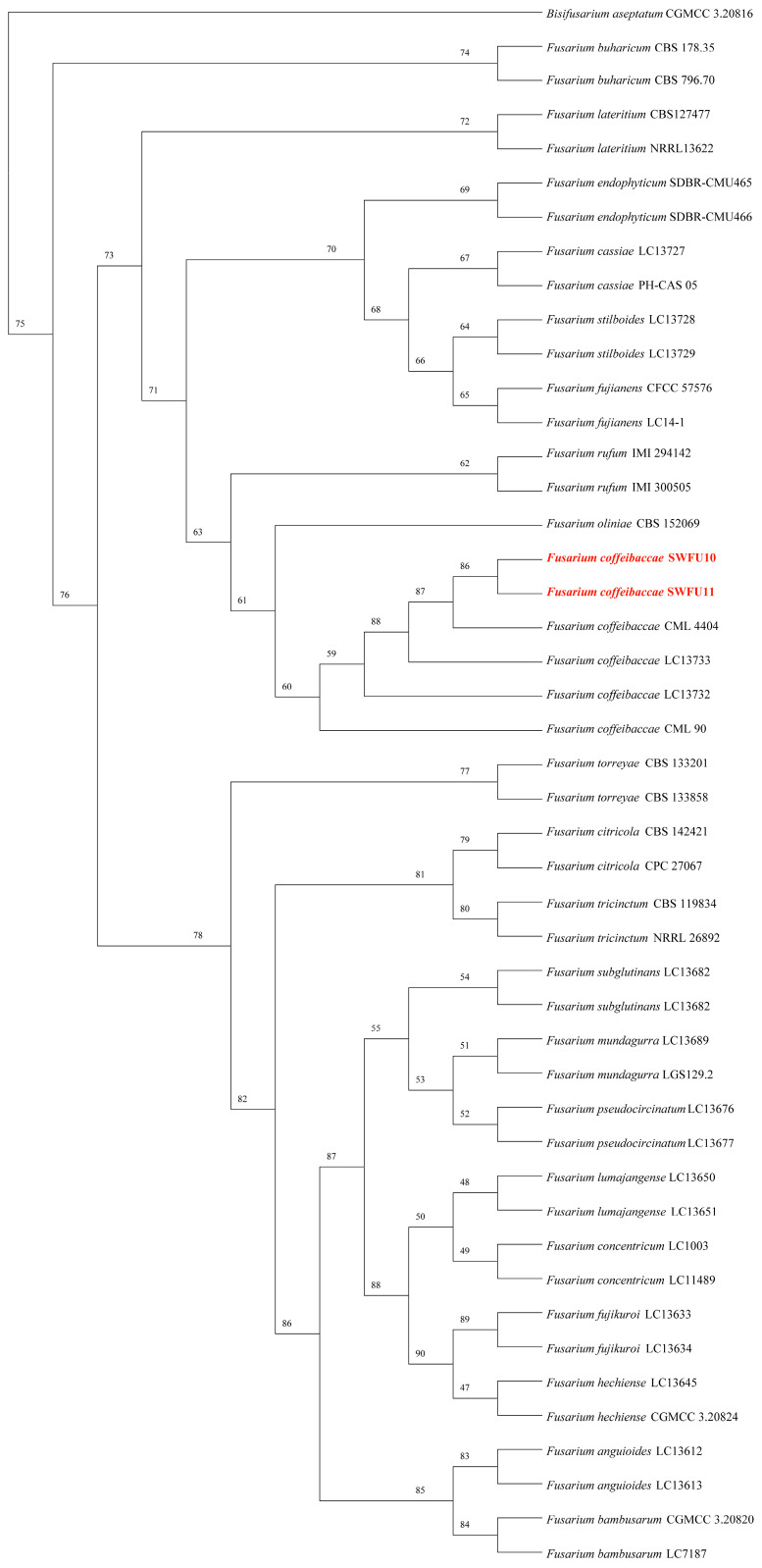
Phylogenetic tree from Bayesian analysis of combined *ITS*, *TEF1-α* and *RPB2* sequences. Newly obtained sequences in this study are in red. The GTR + G nucleotide substitution model was used for the analysis.

The internal transcribed spacer (*ITS*), translation elongation factor 1-α (*TEF1-α*), and RNA polymerase second largest subunit (*RPB2*) partial genes were amplified with the primers ITS4/ITS5, EF1/EF2, 5f2 × 7cr and 7ef × 11ar, respectively. BLAST (https://blast.ncbi.nlm.nih.gov/Blast.cgi, accessed on 13 February 2026) comparison in the NCBI database revealed that the strain (SWFU10) exhibited 99.60%, 100%, and 100% similarity with *F. coffeibaccae* strain LC13733 (MW016560, MW594313, and MW474546), respectively. All sequences were deposited in GenBank as for *ITS* (PV211189 and PV211190), *TEF-1α* (PQ867811 and PQ867812), and *RPB2* (PV261064 and PV261065). For phylogenetic analysis, the generated sequences were aligned with 127 reference sequences representing 22 *Fusarium* spp. retrieved from GenBank. (Table 2). The aligned *RPB2* (1849 bp), *ITS* (451 bp), and *TEF1-α* (661 bp) sequences were concatenated into multilocus dataset. The analysis clearly defines the classification status of *F. coffeibaccae.*, supported by very high statistical confidence. In the tree, all *F. coffeibaccae.* sequences, including the newly obtained red-marked sequence from this study, cluster together as a distinct branch with 100% support, forming a stable, monophyletic group. This result confirms that the molecular identity of all examined strains is *F. coffeibaccae* and demonstrates the high internal sequence consistency of this species. The combined analysis of the three genes provides abundant phylogenetic signal; its high resolution and strong support distinguish *F. coffeibaccae* from closely related species and also supply robust evidence for the species’ accurate molecular identification and its evolutionary position within the genus *Fusarium* (Figure 3). The Bayesian phylogeny of the genus *Fusarium* reveals clear species relationships. All major branches received high posterior probability support (≥0.95), indicating a robust tree structure. Multiple recognized *Fusarium* species complexes are identifiable in the tree. The *F. coffeibaccae* complex occupies a prominent position, showing signs of recent active differentiation and broad ecological adaptation. It comprises six *F. coffeibaccae* samples; although they form a single clade, they display discernible genetic differentiation within (average genetic distance 0.02–0.03) (Figure 4).

### 3.4. Pathogenicity Test

**Figure 5:**
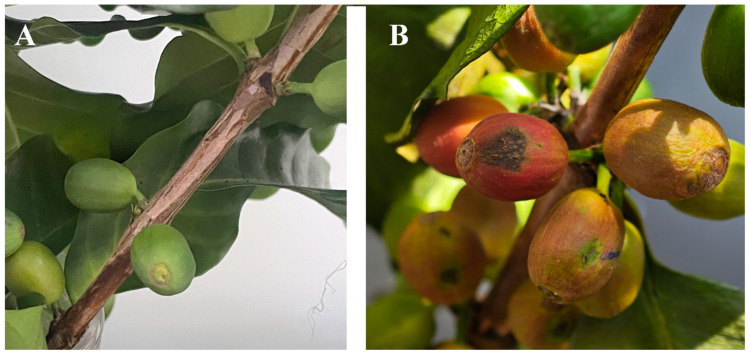
Symptoms of re-inoculated fruit after 17 days. (**A**) Control. (**B**) Infection.

After 17 days of reinoculation, the coffee fruits first reddened, then blackened, shriveled, and remained attached to the branch, while no symptoms were observed in the control group (Figure 5A,B). Re-isolation was successful from 100% of symptomatic fruits (20/20 fruits, three independent batches); no fungus was recovered from control fruits. Colony morphology (aerial white mycelium turning pale violet, abundant micro- and macroconidia, typical conidiophore branching) and *ITS* (PV211190), *TEF-1α* (PQ867812), and *RPB2* (PV261065) of the representative re-isolate SWFU11 were identical to those of the original inoculum SWFU10, thus satisfying Koch’s postulates.

## 4. Discussion

This study successfully isolated 10 strains from diseased coffee fruits collected in Menglian, Yunnan. All isolates were identified as *Fusarium* spp., strongly suggesting that *Fusarium* is the primary pathogen responsible for coffee berry disease in this region. In some earlier studies, Wrigley confirmed the association between *F. lateritium* and *F. xylarioides* with coffee fruit rot disease [25]. Furthermore, in studies conducted in Puerto Rico, five species (*F. bostrycoides*, *F. lateritium*, *F. nirenbergiae*, *F. solani-melongenae*, and *F. pseudocircinatum*) were demonstrated to be pathogenic to coffee fruits [7]. Pathogenicity assays demonstrated that isolate SWFU10 can infect coffee fruits and reproduce typical fruit rot symptoms, thereby fulfilling Koch’s postulates and providing the first confirmed evidence of *Fusarium coffeibaccae* causing coffee fruit rot in China. Previously, Wang et al. [26] isolated two strains, *Fusarium* sp. LC13732 and *Fusarium* sp. LC13733, from *Coffea* sp. in Yunnan Province. These strains were subsequently identified by Costa et al. as *F. coffeibaccae*, suggesting a stable association between this species and coffee in China [19,26]. However, those earlier studies did not evaluate pathogenicity, leaving the causal role of this fungus unresolved. By combining accurate species identification with pathogenicity verification, the present study establishes, for the first time, the pathogenic role of *F. coffeibaccae* in coffee fruit rot in China and clarifies a gap left by previous reports.

*Fusarium coffeibaccae* belongs to the *Fusarium lateritium* species complex (FLSC) and was formally described as a novel species by Costa et al. [19]. Among members of the FLSC, it appears to be one of the most widely distributed and genetically diverse taxa, with reports from Brazil and several African countries. The pathogen infects multiple hosts, including coffee, citrus, and dragon fruit, causing bark diseases and fruit rot [19]. Despite its broad geographic distribution and host range, its epidemiological significance in China’s coffee-producing regions has remained largely unknown. Moreover, studies on *Fusarium*-associated diseases in Chinese coffee plantations are scarce. Considering that other members of the FLSC, including *Fusarium lateritium*, have been associated with severe coffee diseases in other regions, the recovery of *F. coffeibaccae* from diseased berries in this study may indicate a potentially underrecognized threat to coffee production in China.

Although the morphological characteristics of strains SWFU10 and SWFU11 in this study were consistent with the typical descriptions of *F. coffeibaccae*, the observed morphological variations necessitated molecular phylogenetic analysis to provide compelling evidence. With the development of molecular biology, taxonomists have increasingly integrated morphological characteristics with molecular biology for classification purposes. In 1989, Guadet et al. initiated the application of molecular phylogenetic methods [27]. Subsequently, O′Donnell et al. extensively employed these methods and demonstrated that they could better elucidate the relationships among *Fusarium* species compared to single morphological classifications [28]. To address the challenges posed by high species diversity and the limitations of morphological identification, Geiser and Park et al. developed FUSARIUM-ID (https://www.fusarium.org/page/TaxonomySearch accessed on 13 February 2026), a publicly available DNA sequence-based identification platform [29,30,31]. Notably, O'Donnell et al. integrated three gene loci and incorporated 69 clinically relevant Fusarium species associated with human and animal infections, significantly enhancing its utility in clinical pathogen identification [32].)According to the study by Kerry O’Donnell et al. [33], accurate species-level identification of *Fusarium* relies heavily on the use of phylogenetically informative gene fragments, such as translation elongation factor 1-alpha (*TEF1-α*) and the second largest subunit of RNA polymerase II (*RPB2*). Based on this, the present study employed *TEF1* and *RPB2* as the primary molecular markers, supplemented by the *ITS* rDNA region for multigene sequence analysis.

Phylogenetic analysis was performed using a concatenated dataset of *ITS*, *TEF1-α*, and *RPB2* sequences, which included 127 reference sequences representing 22 *Fusarium* taxa. Maximum likelihood analysis revealed that the isolates SWFU10 and SWFU11 clustered within a well-supported clade together with reference strains of *F. coffeibaccae*, including strain LC13733 and culture ex-epitype strain CML 90. The clade received strong bootstrap support, confirming the con-specificity of the examined isolates with *F. coffeibaccae*. The phylogenetic concordance observed across multiple gene regions, along with the high sequence similarity to reference strains, firmly establishes the identity of the isolates SWFU10 and SWFU11 as *F. coffeibaccae*. This study underscores the importance of a multi-locus phylogenetic approach for reliable species delineation in *Fusarium*, particularly in groups where morphological characters alone are insufficient for definitive identification.

The phylogenetic reconstruction not only resolved the taxonomic placement of isolates SWFU10 and SWFU11 within FLSC, but also provided robust multilocus evidence supporting their conspecificity with *F. coffeibaccae* [19,34]. The strong statistical support for the subclade containing our isolates and the ex-epitype strain CML 90ᵀ reinforces the stability of the current phylogenetic species concept within this complex. Given the well recognized taxonomic ambiguity historically associated with morphologically similar members of the FLSC, the integration of multilocus sequence data is critical for accurate species delimitation and epidemiological inference.

Importantly, this study goes beyond a routine pathogen record by clarifying the pathogenic role of *F. coffeibaccae* on coffee fruits under Chinese production systems. Previous reports documented its association with coffee but did not experimentally establish pathogenicity. By fulfilling Koch’s postulates and integrating phylogenetic resolution with pathogenicity verification, our findings substantiate *F. coffeibaccae* as an active etiological agent rather than a secondary colonizer. This distinction is essential for risk assessment and disease management. The detection of *F. coffeibaccae* in Yunnan also raises broader questions regarding host adaptation and geographic expansion within the FLSC. Members of this complex exhibit diverse host ranges and ecological strategies, suggesting evolutionary plasticity. The close phylogenetic affinity of *F. coffeibaccae* to related taxa such as *F. oliniae* and *F. rufum* may indicate recent divergence events linked to host specialization. Understanding whether the emergence of coffee fruit rot in China represents a recent introduction, local host shift, or previously undetected endemic presence warrants population-level genomic investigation.

From a phytosanitary perspective, the globalization of coffee germplasm exchange provides a plausible pathway for pathogen dissemination. The increasing connectivity of agricultural trade networks, coupled with climate variability that may favor fungal infection cycles, creates ecological conditions conducive to disease emergence. Therefore, the occurrence of *F. coffeibaccae* in China should be interpreted within the broader framework of emerging fungal pathogens under global change scenarios. Despite the 100% disease incidence observed under controlled inoculation, field epidemiology remains unresolved. The potential involvement of alternative hosts or crop residues as inoculum reservoirs suggests that disease dynamics may be shaped by landscape-level ecological interactions. Future research integrating population genomics, environmental monitoring, and host–pathogen interaction studies will be essential to elucidate survival strategies, dispersal mechanisms, and virulence determinants.

As the first confirmed pathogenic report of *F. coffeibaccae* on coffee in China, this study provides a molecularly validated baseline for surveillance and management. The development of locus-specific diagnostic tools (*TEF1-α* and *RPB2*-based assays) will facilitate early detection, while large-scale population surveys will clarify distribution patterns and genetic structure. Ultimately, integrating taxonomic resolution with epidemiological and ecological investigations will be crucial for developing sustainable disease management strategies and safeguarding the long-term stability of coffee production in Yunnan.

In conclusion, the present study establishes *F. coffeibaccae* as a confirmed pathogen of coffee fruit in China and situates this finding within a broader evolutionary and biosecurity context. Beyond documenting a new occurrence, our results highlight the importance of multilocus phylogenetics in resolving species complexes and underscore the need for proactive monitoring of emerging fungal pathogens in rapidly developing agricultural systems. Nevertheless, we acknowledge the limitation regarding the geographical and strain sampling scale. Future studies should encompass a broader geographical range and include a larger number of isolates to fully elucidate the population structure, genetic diversity, and pathogenic potential of *F. coffeibaccae* associated with coffee. Such research will be vital for developing sustainable management strategies for this emerging disease. Despite this limitation, the present work establishes a critical foundation by definitively identifying the pathogen and validating its pathogenicity through Koch’s postulates, thereby providing a crucial reference for future phytopathological and epidemiological studies on coffee fruit rot in the region.

## 5. Conclusions

In this study, the pathogen responsible for coffee fruit rot in Yunnan was conclusively identified as *F. coffeibaccae* based on an integrative taxonomic approach combining detailed morphological characterization, multilocus phylogenetic analyses (*ITS*, *TEF1-α*, and *RPB2*), and pathogenicity assays fulfilling Koch’s postulates. This study therefore constitutes the first confirmed report of *F. coffeibaccae* causing coffee fruit rot in China, significantly expanding the known geographic distribution of this species into Chinese coffee-producing systems. Beyond documenting a new regional occurrence, our findings provide a molecularly validated baseline for epidemiological surveillance, risk assessment, and the development of targeted disease management strategies for coffee fruit rot in Yunnan.

## Figures and Tables

**Table 1:** Primer sequences.

Locus	Gene Product	Primer	Sequence (5′-3′)	Reference
*TEF1-α*	Translation elongation factor 1α	EF1	ATGGGTAAGGARGACAAGAC	[20]
		EF2	GGARGTACCAGTSATCATG	
*RPB2*	RNA polymerase second largest subunit	RPB2-5F2	GGGGWGAYCAGAAGAAGGC	[21]
		FRPB2-7CR	CCCATRGCTTGYTTRCCCAT	
		FRPB2-7CF	ATGGGYAARCAAGCYATGGG	[22]
		RPB2-11AR	GCRTGGATCTTRTCRTCSACC	
*ITS*	Internal transcribed spacer region of the nrDNA	ITS4	TCCTCCGCTTATTGATAT	[23]
		ITS5	GGAAGTAAAAGTCGTAACA	

**Table 2:** GeneBank accession numbers of the sequences used in the phylogenetic analyses.

Isolate	Country	Host	GenBank Accession Number		
			*ITS*	*TEF1-α*	*RPB2*
*F. anguioides*					
LC13612 = M0563	China	*Cordyline stricta*	MW016395	MW580435	MW474381
LC13613 = M0568	China	*Alocasia odora*	MW016396	MW580436	MW474382
*F. bambusarum*					
CGMCC 3.20820 = LC7180 T	China	*Bambusoideae*	MW016403	MW580443	MW474389
LC7187	China	*Bambusoideae*	MW016404	MW580444	MW474390
*F. concentricum*					
LC1003	China	*Reineckia carnea*	MW016409	MW580449	MW474395
LC11489 = G1	China	*Vitis* sp.	MW016410	MW580450	MW474396
*F. fujikuroi*					
LC13633 = F013	USA	*Glycine max*	MW016432	MW580472	MW474418
LC13634 = F032	Japan	*Acer palmatum*	MW016433	MW580473	MW474419
*F. hechiense*					
CGMCC 3.20824 = LC13644 = GXHCSWL14-E1 T	China	*Musa nana*	MW016454	MW580494	MW474440
LC13645 = GXHCSWL14-E12	China	*Musa nana*	MW016455	MW580495	MW474441
*F. lumajangense*					
LC13650 = GXCZMQF02-1	China	*Musa nana*	MW016461	MW580501	MW474447
LC13651 = GXCZMQF02-2	China	*Musa nana*	MW016462	MW580502	MW474448
*F. mundagurra*					
LC13689 = LGS129	China	*Paspalum vaginatum*	MW016516	MW580556	MW474502
LGS129.2	China	*Paspalum vaginatum*	MZ379241	MZ399211	MZ399208
*F. pseudocircinatum*					
LC13676 = F428	China	*Syzygium samarangense*	MW016502	MW580542	MW474488
LC13677 = F429	China	*Syzygium samarangense*	MW016503	MW580543	MW474489
*F. subglutinans*					
LC13682 = F055	USA	*Glycine max*	MW016509	MW580549	MW474495
LC13683 = F057	USA	*Zea mays*	MW016510	MW580550	MW474496
*F. lateritium*					
CBS127477	China	*Twig*	OR538707	PQ000517	PQ000681 PQ000759
NRRL13622	USA	*Ulmus* sp.	OR538706	PQ000518	JX171571
*F. stilboides*					
LC13728 = CQ1109	China	*Forsythia* sp.	MW016555	MW594308	MW474541
LC13729 = CQ993	China	*Forsythia* sp.	MW016556	MW594309	MW474542
*F. cassiae*					
LC13727 = F092	China	*Coffea* sp.	MW016554	MW594307	MW474540
PH-CAS 05	Thailand	*Cassia fistula*	MT215495	MT212205	PX130470
*F. endophyticum*					
SDBR-CMU465	Thailand	*Camellia sinensis*	OQ683543	OQ686616	PQ000677 PQ000755
SDBR-CMU466	Thailand	*Camellia sinensis*	OQ683544	OQ686617	PQ000678 PQ000756
*F. fujianense*					
CFCC 57576 = LC14	China	*Cunninghamia lanceolata*	ON564582	ON734389	PX130467
LC14-1	China	*Cunninghamia lanceolata*	ON564583	ON734390	PX130468
*F. buharicum*					
CBS 178.35 = DSM 62166 = IMB 11176 = NRRL 25488	Uzbekistan	*Gossypium herbaceum*	MH855630	KX302912	KX302928
CBS 796.70 = ATCC 24135 = BBA 11122 = DSM 62165 = FRC R-4955 = IMB 11122 = NRRL 13371	Iran	*Hibiscus cannabinus*	OM117592	OM160859	OM160838
*F. coffeibaccae*					
CML 90^T^ = CBS 152040 = CPC 46658	Brazil	*Coffea arabica*	-	PQ000464	PQ000629 PQ000707
CML 4404 = CBS 152043 = CPC 46663	Brazil	*Coffea arabica*	-	PQ000468	PQ000633 PQ000711
SWFU10	China	*Coffea arabica*	PV211189	PQ867811	PV261064
SWFU11	China	*Coffea arabica*	PV211190	PQ867812	PV261065
LC13732 = F085	China	-	MW016559	MW594312	MW474545
LC13733 = F088	China	-	MW016560	MW594313	MW474546
*F. torreyae*					
CBS 133201 = MAFF 243466 = NRRL 54149 = 5005-08 Canker = Path lab	USA	*Torreya taxifolia*	HM068344	HM068337	JX171548
CBS 133858 = MAFF 243468 = NRRL 54151	USA	*Torreya taxifolia*	HM068346	HM068339	OM160857
*F. citricola*					
CBS 142421 = CPC 27805	Italy	*Citrus reticulata*	LT746245	LT746197	LT746310
CPC 27067	Italy	*Citrus limon*	LT746242	LT746194	LT746307
*F. tricinctum*					
CBS 119834 = FRC R-9978 = KSU 11441 = MRC 8381 = NRRL 53913 = Univ. Sydney F12844	Unknown	Unknown	OL832259	OL772831	OL773135
NRRL 26892 = 93101 = a43	Finland	*Hordeum vulgare*	OL832254	OL772826	OL773130
*F. oliniae*					
CBS 152069 = CPC 38826^T^	South Africa	*Olinia* sp.	-	PQ000522	PQ000685 PQ000763
*F. rufum*					
IMI 294142 = CPC 42977	India	*Citrus medica*	-	PQ000523	PQ000686 PQ000765
IMI 300505^T^ = CPC 43032	India	*Parkia* sp.	-	PQ000524	PQ000687 PQ000766
*Bisifusarium aseptatum*					
CGMCC 3.20816 = LC1075 T	China	*Orchidaceae* sp.	MW016389	MW580429	MW474375

## Data Availability

The data presented in this study are openly available in NCBI at https://static.pubmed.gov/portal/portal.fcgi/ accessed on 13 February 2026.
